# EPIC-DB: a proteomics database for studying Apicomplexan organisms

**DOI:** 10.1186/1471-2164-10-38

**Published:** 2009-01-21

**Authors:** Carlos J Madrid-Aliste, Joseph M Dybas, Ruth Hogue Angeletti, Louis M Weiss, Kami Kim, Istvan Simon, Andras Fiser

**Affiliations:** 1 Biodefense Proteomics Research Center, Albert Einstein College of Medicine, 1300 Morris Park Avenue, Bronx, NY 10461, USA; 2 Department of Systems and Computational Biology, Albert Einstein College of Medicine, 1300 Morris Park Avenue, Bronx, NY 10461, USA; 3 Department of Biochemistry, Albert Einstein College of Medicine, 1300 Morris Park Avenue, Bronx, NY 10461, USA; 4 Department of Pathology, Albert Einstein College of Medicine, 1300 Morris Park Avenue, Bronx, NY 10461, USA; 5 Department of Developmental and Molecular Biology, Albert Einstein College of Medicine, 1300 Morris Park Avenue, Bronx, NY 10461, USA; 6 Department of Medicine, Albert Einstein College of Medicine, 1300 Morris Park Avenue, Bronx, NY 10461, USA; 7 Department of Microbiology and Immunology, Albert Einstein College of Medicine, 1300 Morris Park Avenue, Bronx, NY 10461, USA; 8 Laboratory for Macromolecular Analysis and Proteomics, Albert Einstein College of Medicine, 1300 Morris Park Avenue, Bronx, NY 10461, USA; 9 Albert Einstein College of Medicine, 1300 Morris Park Avenue, Bronx, NY 10461, USA; 10 Institute of Enzymology, Hungarian Academy of Sciences, Karolina ut 29, Budapest 1113, Hungary

## Abstract

**Background:**

High throughput proteomics experiments are useful for analyzing the protein expression of an organism, identifying the correct gene structure of a genome, or locating possible post-translational modifications within proteins. High throughput methods necessitate publicly accessible and easily queried databases for efficiently and logically storing, displaying, and analyzing the large volume of data.

**Description:**

EPICDB is a publicly accessible, queryable, relational database that organizes and displays experimental, high throughput proteomics data for *Toxoplasma gondii *and *Cryptosporidium parvum*. Along with detailed information on mass spectrometry experiments, the database also provides antibody experimental results and analysis of functional annotations, comparative genomics, and aligned expressed sequence tag (EST) and genomic open reading frame (ORF) sequences. The database contains all available alternative gene datasets for each organism, which comprises a complete theoretical proteome for the respective organism, and all data is referenced to these sequences. The database is structured around clusters of protein sequences, which allows for the evaluation of redundancy, protein prediction discrepancies, and possible splice variants. The database can be expanded to include genomes of other organisms for which proteome-wide experimental data are available.

**Conclusion:**

EPICDB is a comprehensive database of genome-wide *T. gondii *and *C. parvum *proteomics data and incorporates many features that allow for the analysis of the entire proteomes and/or annotation of specific protein sequences. EPICDB is complementary to other -genomics- databases of these organisms by offering complete mass spectrometry analysis on a comprehensive set of all available protein sequences.

## Background

High throughput proteomics experiments (mass spectrometry) provide unique insight into the protein expression profile of an organism and can be useful in identifying the correct gene structure of a genome, particularly when splice variants or alternative gene predictions exist, or to elucidate the roles of post-translational modifications within proteins [1]. Indeed, large scale proteomics approaches have been used in recent years to analyze genomes of various organisms such as *S. cerevisiae *[2], *M. mobile *[3], *C. parvum *[4], *T. gondii *[5], and *S. luteogriseus *[6], as well as various subproteomes [7-9]. Recent advances in high throughput methods as well as the increased emphasis on and amount of high throughput data necessitate publicly accessible and easily queried databases for efficiently storing, displaying, and analyzing the large volume of data. We have established a proteomics database, EPICDB (Experimental ProteomICs Database) to accommodate experimental data from the large-scale proteomics exploration of two Apicomplexan organisms; *Toxoplasma gondii *and *Cryptosporidium parvum*.

*T. gondii *is an obligate intracellular protozoan, belonging to the phylum Apicomplexa, and is an important pathogen in both immune competent and immune compromised humans. The parasite causes chronic infection in adults and is present in an estimated 22.5% of people older than 12 in the United States [10] and up to 90% of the population in other regions of the world [11]. *T. gondii *clinical disease is most typical in immune compromised individuals and congenitally infected children and is a common opportunistic pathogen associated with AIDS. Also, *T. gondii *is an important model system for the phylum Apicomplexa [12], which includes, among others, Plasmodium (malaria) species. Unlike many other Apicomplexa, which are experimentally intractable, *T. gondii *is easily cultured in *vitro*, has well established experimental protocols for genetic manipulation, and has a well characterized mouse model [13]. *Cryptosporidium parvum *is an Apicomplexan parasite that infects the epithelial cells of the microvillus border of the gastrointestinal tract where it resides in a unique vacuole bellow the host cell membrane but outside the host cell cytoplasm [14]. This organism forms resistant oocysts which are transmitted effectively by food or water. Ingestion of these oocysts results in infection of the gastrointestinal track with the development of diarrhea, which can be profuse especially in immune compromised hosts. Unlike *T. gondii*, *in vitro *culture is difficult and there are no established experimental protocols for genetic manipulation. Due to waterborne outbreaks associated with *T. gondii *[15] and *C. parvum *[16], these organisms are classified by the National Institute of Allergy and Infectious Diseases as Category B priority pathogens.

EPICDB contains high throughput mass spectrometry data that is cross referenced to all available computationally and experimentally derived protein sequences for *T. gondii *and *C. parvum*. The database can be queried to find proteins that are experimentally verified by proteomics data and, in doing, can aid in the discovery of sequences that have not been fully annotated but are supported by experimental data. Further, since all the proteomics data are comprehensively searched against all available protein sequences, EPICDB is a good resource for assessing discrepancies among sequences predicted by different algorithms and for examining splice variants of the same gene. Additionally, the format is suitable to accommodate additional experimental data or other organisms and can be used as a model system for other genomes for which high throughput proteomics data will become available.

The primary focus of EPICDB is to provide a comprehensive proteome-based description of the selected pathogens. This distinguishes it from other, genome-based databases, which exist for various apicomplexan and other organisms, such as, for example, ToxoDB , CryptoDB , or TrichDB .

## Construction and content

### Database construction

EPICDB is a relational database using a MySQL database management system and consisting of 40 tables. Within the database, query-response time is optimized by reading a summary table containing the results of a complex "JOIN" query.

The CGI::Application  framework was used for the web application development. CGI::Application is a stable and lightweight open source framework for building web-applications on any operating system that supports Perl and CGI. It encourages developers to adopt a model-view-controller (MVC) architecture, which separates the application logic that interacts with a database (model) from the HTML pages presented to the client (view) and the user input actions (controller). At the front end, HTML pages, CSS files, and Javascript code are implemented using a template system (HTML::Template).

### Database content

EPICDB is a collection of experimental and computational data that are referenced to all available protein sequences for *T. gondii *and *C. parvum*, which represent the theoretical proteomes of the respective organisms. The available *T. gondii *protein sequences were compiled from five datasets; TigrScan [17], TwinScan [18], GlimmerHMM [17], ToxoDB.org Release4, and experimental sequences from the NCBI protein databases. TigrScan, TwinScan, and GlimmerHMM are computational gene finder algorithms that were employed by The Institute for Genomic Research (now the J. Craig Venter Institute) on the ME49 strain of *T. gondii*. The Release4 dataset is an annotation of the ME49 strain with sequences predicted by the GLEAN [19] algorithm. The dataset of experimental sequences was obtained from the NCBI Entrez Protein Database, which was filtered, by the organism name, for *Toxoplasma gondii *(As of July 2008, predicted genes/proteins from *T. gondii *genome analysis have not been deposited to NCBI or GenBank databases). The five datasets were combined to provide a comprehensive set of all available protein sequences for the *Toxoplasma gondii *proteome and comprised 30,197 sequences. While *T. gondii *has an intron rich genome and, consequently, presents substantial difficulty for gene prediction programs to properly identify gene coding regions, in *C. parvum *there are almost no introns and gene prediction is rather straightforward [20]. The available *C. parvum *sequences were obtained from CryptoDB  and from the NCBI Entrez Protein Database, which was filtered, by the organism name, for *Cryptosporidium Parvum*. The combined *C. parvum *dataset comprises 8,316 protein sequences. In the case of both organisms the sequence sets are redundant due to computational algorithms predicting the same (or essentially same) sequences or the same sequences appearing more than once as experimentally derived sequences in the NCBI database.

Because of the redundancy among the alternate gene models, the database is organized in clusters of the protein sequences that share at least 90% sequence identity for the overlapping parts [21]. Clustering allows the user to evaluate redundancy or prediction differences among different sequences. However, since the clustering scheme allows sequences to be grouped if they share local sequence similarity and not only sequences that are exact matches for the entire length, in many cases, clustering also offers the possibility of exploring alternative splicing events in the genome, when proteins are predicted from the same genomic location and differ by the inclusion, exclusion, elongation, or truncation of some introns or exons. The *T. gondii *dataset contains 30,197 alternative protein sequences, or possible splice variants, that are clustered into 14,983 groups of sequences, or possible protein coding genomic regions. The *C. parvum *dataset contains 8,316 sequences that are clustered into 3,852 groups.

EPICDB currently contains the results of 183 high throughput, tandem mass spectrometry experiments that were performed on *T. gondii *plasma membrane, cytoskeletal, cyst wall, and cytosolic protein preparations. These experiments produced 33,045 proteolytic peptides that, when searched against a database of the *T. gondii *protein sequences, experimentally verified 8,372 sequences (that can be grouped into 3,233 clusters). Further, EPICDB contains data from 88 high throughput, tandem mass spectrometry experiments that were performed on *C. parvum*. These experiments produced 3,049 proteolytic peptides that experimentally verified 1,241 *C. parvum *protein sequences (481 clusters). The data contained within EPICDB is a comprehensive summary of the mass spectrometry experiments and the MASCOT  searches of the data against the theoretical proteomes. For each protein, a list of the experiments that identified a proteolytic peptide that maps to that protein is included, along with the type of cell fraction analyzed, the type of mass spectrometry experiment conducted, and the number of peptide hits that were obtained. The mass spectrometry data and the results of the MASCOT search are provided and publicly available in five formats; CSV (comma separated value) formatted mass spectrometry files, RAW unformatted mass spectrometry data files, MGF (MASCOT Generic Format) and DTA files, containing peak lists of mass and intensity values, and MASCOT search summary files. Data for peptide scores, expectation values, location in the protein, and protein coverages are included for each proteolytic peptide that is mapped to the protein.

As an example of the addition of other types of experimental data, EPICDB also currently stores the results of antibody experiments that were performed on 52 *T. gondii *and 34 *C. parvum *protein sequences, including information on the peptide sequences used for immunization in the production of the antibodies, the amount of sera produced for each peptide mixture, and the results of Enzyme-Linked ImmunoSorbent Assays (ELISA), Immuno-Fluorescence Assays (IFA), and Immunoblot experiments, with images if available.

Additional mass spectrometry and antibody experiments are being continuously performed by the Albert Einstein Biodefense Proteomics Research Center and the results are incrementally added to EPICDB.

EPICDB contains functional annotations and comparative genomics for the theoretical proteomes of T. *gondii *and C. *parvum*. Transmembrane segments and signal peptides were predicted using the Phobius program [22]. All known PFAM domains [23] were also identified. For each sequence in EPICDB, orthologous proteins were identified from sequences in the human genome, in other Apicomplexan genomes, and for the complete NCBI non-redundant protein database. Aligned genomic data (cDNA (ESTs) and ORFs) are also presented for each *T. gondii *protein sequence within the database. EST sequences were obtained from the NCBI EST "others" database and filtered for the organism name. ORF sequence data was obtained from ToxoDB.org version Release4. All experimental details and analysis of how mass spectrometry peptide hits validate alternative gene predictions can be found in a separate research article (J. Dybas et al. Computational Analysis and Experimental Validation of Gene Predictions in Toxoplasma gondii PLOSone. 2008;3(12):e3899. Epub 2008).

## Utility and discussion

### Querying the database

**Figure 1:**
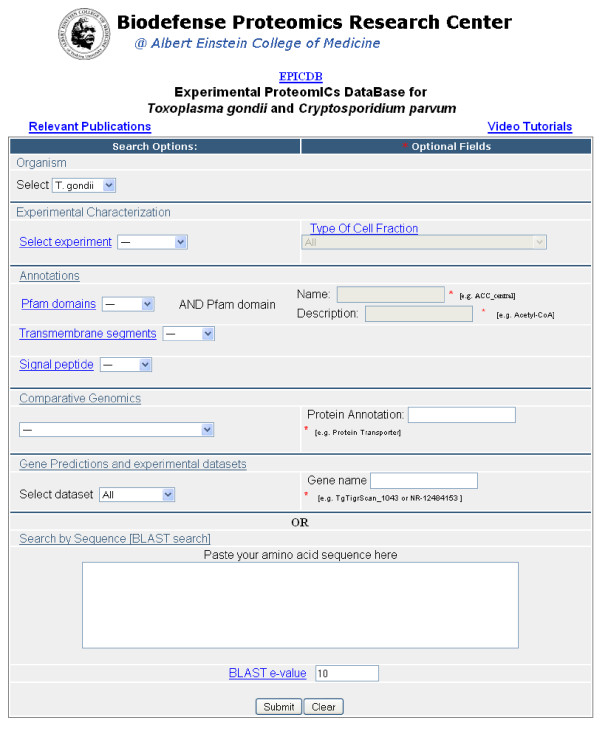
**Screenshot of the EPICDB query front page**. This page is where the user chooses the organism to be studied and queries the database for "Experimental Characterizations", "Annotations", "Comparative Genomics", or "Gene predictions and experimental datasets" or searches the database for a specific sequence.

EPICDB is accessed through a query front-page where the user can define conditions for searching the underlying relational database (Figure 1). The minimum parameter that must be set is the type of genome to be searched (*Organism*). If no other parameters are specified all possible protein sequences will be listed and clustered.

The database can be queried in a variety of ways. The *Experimental Characterization *query option selects the subset of protein sequences that are experimentally validated by mass spectrometry and/or antibody data. For the mass spectrometry experiments it is possible to select a subset of proteins based on the type of experimental cell fraction, such as membrane, cytosolic, or deglycosylated cell wall. The *Annotations *query is used to search for those proteins that are functionally characterized in terms of transmembrane domains, signal peptides and/or Pfam domains. Within the Pfam domains, it is possible to search by a specific domain name, such as "ACC_central", or by a description such as "Acetyl-CoA". The *Comparative Genomics *query locates predicted proteins that share a detectable sequence similarity (based on a BLAST search [24]) with other Apicomplexan genomes, with the human genome, or with any known proteins (referencing against the complete NCBI non-redundant protein database). Annotations from similar proteins are inherited and stored for each EPICDB protein sequence and the database search can be refined by a search for specific keywords, such as "transporter". Any or all of the aforementioned parameters can be selected to be combined in a single query.

In addition to seeing the query results for the entire theoretical genome, the *Gene Predictions and Experimental Datasets *query allows for the selection of proteins based on the type of gene prediction method or by only experimentally derived proteins. Within this query option one can search the database for a specific gene name, such as "TgTigrScan_1043".

Rather than querying the database based on experimental characteristics, it is possible to perform a BLAST search for a user-input protein sequence, or sequence fragment, against the entire EPICDB. The sensitivity of the BLAST search can be adjusted by defining the corresponding e-value cutoff.

### Parsing the query results

**Figure 2:**
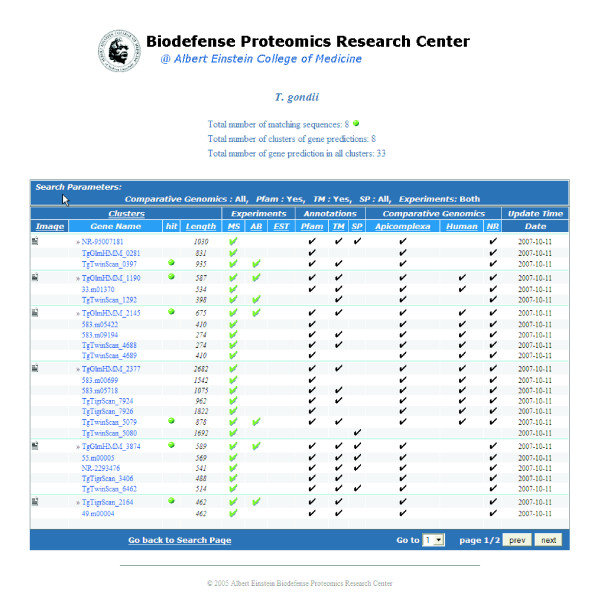
**Screenshot of the query results page**. The sequence clusters that contain a protein matching the user-defined query are displayed along with a summary of their corresponding experimental and computational data.

The results page (Figure 2) displays the clusters of sequences where at least one of the cluster members matches the user-defined query. The top of the query results page lists the number of sequences that match the query criteria, the number of clusters that those sequences belong to, and the number of all the sequences within the matching clusters.

**Figure 3:**
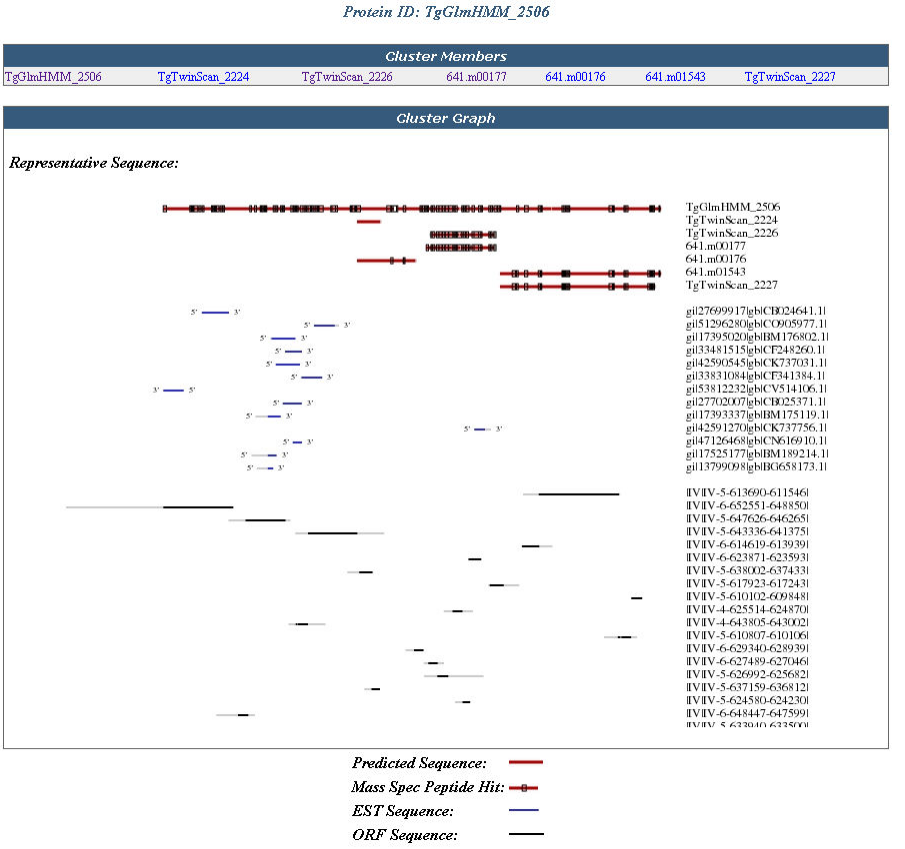
**Sequence cluster image**. The cluster image showing a cluster of protein sequences (red line) and assigned mass spectrometry peptides (black boxes on protein sequences). Below the protein sequences are the aligned ESTs (blue lines with directionality indicated) and ORFs (black lines). A unique identifier is included for each protein, EST, and ORF sequence.

The query results table lists the protein sequences and a summary of their corresponding experimental and computational data. The header of the results table displays the search parameters that were used to query the database. The clusters of proteins are listed and the sequences are displayed using the unique identifiers that were assigned by the prediction algorithms. A green dot next to a sequence indicates that the sequence matched the query criteria. To the left of each cluster there is a link to access an image that shows the aligned protein sequences in the cluster with any mapped mass spectrometry proteolytic peptides along with any aligned EST and ORF sequences (Figure 3). The sequence length is provided for each sequence. In the subsequent columns, to the right of each protein sequence, a check-mark indicates the presence of information on mass spectrometry experiments, antibody production experiments, EST alignments, functional annotations (PFAM domains, transmembrane segments, and signal peptides), or comparative genomics (orthologs found in an Apicomplexan genome, human genome, or the NCBI protein database) for that sequence. Clicking on the checkmark navigates to a subpage where the specific information for that data-type is displayed.

### Viewing experimental and computational data

**Figure 4:**
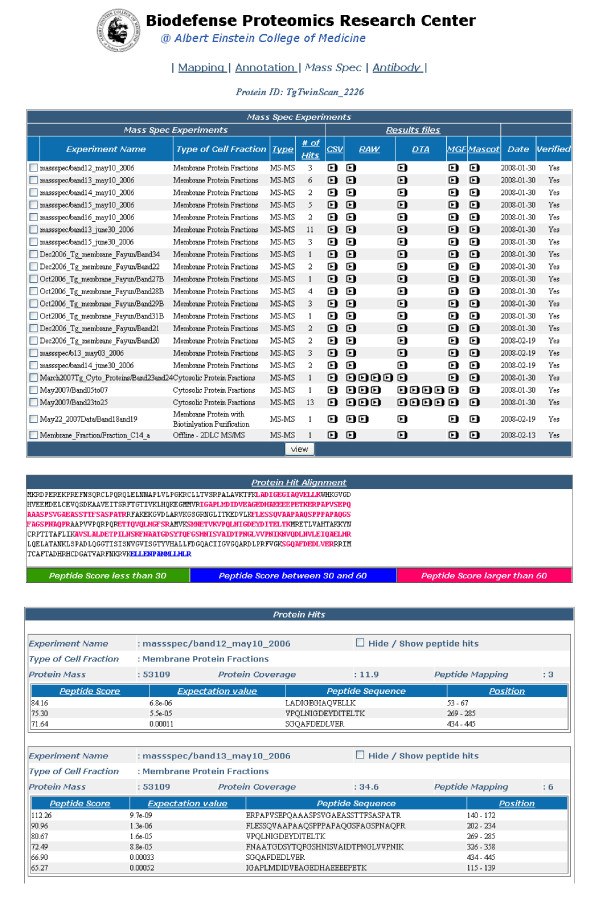
**Screenshot of the "MassSpec" data page**. The mass spectrometry data page contains the data corresponding to all of the mass spectrometry experiments that contained a mass spectrometry peptide that was assigned to the respective protein.

If a specific protein or data-type is selected a protein specific page is displayed with a header containing four options; *Mapping*, *Annotations*, *MassSpec *and *Antibody*. The *Mapping *page (the default destination when a protein is selected) displays the protein sequence length, the actual amino acid sequence, and information on any orthologous proteins in other databases. The *Annotation *page provides a graphical display of the protein sequence and any predicted transmembrane segments, signal peptides, or PFAM domains and, optionally, more specific data for each of the annotation features. The *MassSpec *page (Figure 4) begins with a list of all experiments that identified a relevant proteolytic peptide that maps to the selected protein, along with the type of cell fraction analyzed, the type of mass spectrometry experiment conducted, and the number of peptide hits for the experiment. The mass spectrometry and MASCOT data files (see Database Content) are available in this table. In the section below, the identified proteolytic peptides are mapped on the protein sequence using a color coding scheme that indicates the confidence (MASCOT peptide score) of each peptide. Finally, a more comprehensive analysis of each mass spectrometry experiment is displayed, which includes the protein total coverage by the identified peptides, and the peptide score, expectation value, sequence, and location in the protein, for each identified peptide. The *Antibody *page displays information on antibody production data as well as the sequence of the antibody, the amounts of the sera in different animals, and ELISA, IFA, and Immunoblot experimental results, with images where available.

### Finding help

Most options in the query page contain a link to a help page with a brief description of the specific parameter or option and, where applicable, a link to an associated web page. In addition, most navigating options, category headings, and table titles, on the query page, results page, and data pages contain pin-point helps providing a brief explanation of that entity. The upper right corner of the front (query) page has a link to three Flash formatted video tutorials that describe the options for querying the database and for understanding the query results page and the various data pages.

## Discussion

All available protein sequences for *T. gondii *and *C. parvum *that emerged from various gene prediction approaches or were experimentally derived are compiled into a hypothetical proteomes for each of the respective organisms in order to be able to search the proteomics data with the most comprehensive and unbiased dataset possible. Therefore, while some redundancy is obviously introduced, it also allows for the experimental verification and, potentially, the added scrutiny and study of many more sequences than what would be possible if only one set of protein predictions were adequately searched with the proteomics data.

EPICDB's functionality provides the options for a systems-level analysis as well as specific protein-level analysis. The proteome can be searched for any sequences that have experimental validation. Subsets of sequences can be found for those containing desired characteristics such as functional annotations or comparative genomics. Thus, the proteome can be studied by isolating specific sets of sequences that are of unique interest to the user. Within the obtained sequence sets, individual sequences can be analyzed. The individual sequence can be compared to similar sequences, via the clustering analysis, to determine any possible splice variability of the protein or instances in which there are disparities in the sequence predictions. Proteomics data can be analyzed to assess the validity of the predictions and/or splice variants by studying the distribution of assigned peptides on the amino acid sequence. In some cases a mass spectrometry peptide can indicate the possibility of an additional exon or the presence of incorrect splicing. Finally, EPICDB allows for the user to enter an amino acid sequence of interest and find any similar sequences in the database. This functionality provides the opportunity to examine any experimental evidence for a specific gene that the user is attempting to study and characterize.

At the present time the types of data contained within EPICDB are mass spectrometry and antibody experiments, EST and ORF sequences, functional annotations, and comparative genomics. However, the database can be easily expanded. We are currently planning to integrate genome-wide microarray expression data and ChIP-on-chip data into the database. Also, with little effort, other genomes can be added to the system.

While EPICDB provides a variety of automated annotations and all validation information, one of the major challenges is to properly annotate and analyze predicted genes and proteins on an individual basis. This type of work is best approached by a community effort where various groups have specific areas of expertise, perhaps regarding a specific protein or groups of proteins. Therefore we plan to establish a wiki-like option so experts can add information on annotations or functions of proteins.

## Conclusion

High throughput mass spectrometry data has a variety of important applications to genomics and proteomics. Vast amount of genomic sequence data with comparatively small amounts of experimentally derived protein sequence data necessitate the need for computational gene finders and high throughput mass spectrometry data can inform and support these predictions. High throughput mass spectrometry is an emerging efficient method toward a comprehensive, proteome-wide analysis of an organism, which is an important step in identifying protein interactions, studying protein expression levels, elucidating alternative splice sites, or predicting potential chemotherapeutic targets. EPICDB is a comprehensive database for organizing, querying, and displaying proteome-wide proteomics data and incorporates many features that allow for the analysis of the entire proteomes and/or annotation of specific protein sequences for characterizing the proteomes of *T. gondii *and *C. parvum*.

## Availability and requirements

EPICDB can be accessed by the website . The database and all data is publicly available. There are, on average, 6 unique users per day (statistics do not count access from Albert Einstein College of Medicine computers) and 36.4 Mb of data transferred.

## Authors' contributions

CMA performed the main programming tasks. JMD performed programming tasks and wrote the article. RHA provided mass spectrometry data. LMW provided antibody and mass spectrometry data. KK and IS consulted on the project and curated the database. AF supervised the project.
